# Structure and tree species composition in different habitats of savanna used by indigenous people in the Northern Brazilian Amazon

**DOI:** 10.3897/BDJ.5.e20044

**Published:** 2017-09-28

**Authors:** Rodrigo Leonardo Costa de Oliveira, Hugo Leonardo Sousa Farias, Ricardo de Oliveira Perdiz, Veridiana Vizoni Scudeller, Reinaldo Imbrozio Barbosa

**Affiliations:** 1 State University of Roraima, Boa Vista, Brazil; 2 Federal University of Roraima, Boa Vista, Brazil; 3 National Institute for Research in Amazonia, Manaus, Brazil; 4 Federal University of Amazonas, Biological Sciences Institute, Manaus, Brazil; 5 National Institute for Research in Amazonia, Boa Vista, Brazil

**Keywords:** Floristic survey, species richness, *lavrado*, forest environment, non-forest environment.

## Abstract

**Background:**

Woody plant diversity from the Amazonian savannas has been poorly quantified. In order to improve the knowledge on wood plants of these regional ecosystems, a tree inventory was carried out in four different habitats used by indigenous people living in the savanna areas of the Northern Brazilian Amazon. The habitats were divided into two types (or groups) of vegetation formations: forest (riparian forest, forest island, and *buritizal* = *Mauritia* palm formation) and non-forest (typical savanna). The inventory was carried out in two hectares established in the Darora Indigenous Community region, north of the state of Roraima.

**New information:**

The typical savanna is the most densely populated area (709 stems ha^-1^); however, it has the lowest tree species richness (nine species, seven families) in relation to typical forest habitats: riparian forest (22 species, 13 families and 202 stems ha^-1^), forest islands (13 species, 10 families and 264 stems ha^-1^), and *buritizal* (19 species, 15 families and 600 stems ha^-1^). The tree structure (density and dominance) of the forest habitats located in the savanna areas studied in this work is smaller in relation to forest habitats derived from continuous areas of other parts of the Amazon. These environments are derived from Paleoclimatic fragmentation, and are currently affected by the impact of intensive use of natural resources as timberselective logging and some land conversion for agriculture.

## Introduction

The Amazon is recognized as one of the world's region with the greatest biological diversity, with estimates of up to 16,000 tree species (Steege 2013). The entire region consists of a mosaic of different climates, topographical reliefs, hydrological cycles and soils, which drives the formation of a wide range of vegetation types associated with landscapes, which are home to many different species; many still unknown (Hopkins 2007).

Within this large and complex Amazonian landscape, woody resources are widely used by both indigenous people and riverine communities, especially as timber (Miller et al. 1989, Milliken et al. 1992, Demarchi 2014). Human communities living in forest environments have greater biological diversity and availability of woody-based resources when compared with those inhabiting living in areas with open vegetation, such as the extensive areas of savanna in northern Amazon. This distinction is based on the fact that continuous forest areas are richer in tree species, and have greater biological diversity than those found in forest fragments within savanna ecosystems.

The savanna region of the state of Roraima is the largest savanna area of the Brazilian Amazon, occurring in the northern state of Roraima (Barbosa et al. 2007; Araújo et al. 2017). The savanna area of Roraima is locally referred as *lavrado*, “*campos do rio Branco*" or "*campos de Roraima*", and covers an area of over 40,000 km^2^ within the large Rio Branco-Rupununi landscape complex, which extends into the Republic of Guyana and Venezuela (Barbosa and Fearnside 2005, Barbosa and Campos 2011). Several indigenous lands are found within this large area of savanna. Among them, Raposa/Serra do Sol and São Marcos Indigenous Lands are prominent due to their large size.

The São Marcos Indigenous Land (TISM) comprises 654,110 ha and has 42 indigenous communities (Makuxi, Taurepang and Wapixana ethnicities). Most indigenous practices make use of the available woody plant resources in different habitats of the savanna matrix where these communities live. Since the savanna of the state of Roraima is formed by two large vegetation groups – forest and non-forest (Eden 1970, Santos et al. 2013, Barbosa et al. 2007), the tree species composition and the number of stems available to indigenous communities varies depending on the habitat. Recognizing and valuing these natural resources used by indigenous people in the Amazonian savannas improve the capacity to plan and understand the most appropriate forms of management of woody plant diversity in these areas, which are so poorly studied.

The aim of this study is to make available data of woody plants (trees and shrubs) from forest and non-forest formations used by indigenous communities of the Savanna Area of Roraima, Northern Brazilian Amazon, in order to increase the knowledge on species composition and structure of such environments. The Shannon diversity index and Pielou eveness were calculated and the results compared to other studies in Amazonian savanna areas.

## Project description

### Title

Use and conservation of plant resources in indigenous communities in the north of the state of Roraima, Northern Amazon.

### Study area description

The study area is located in the Darora Community, a Makuxi ethnicity group living within the São Marcos Indigenous Land (3°10'42"N and 60°23'34"W; lat/long - DATUM WGS84), which is at approximately 90 km from the state capital, Boa Vista, northbound on the BR -174 and RR-319 highways, by the Uraricoera river ferry crossing. Based on data from the Boa Vista weather station, the climate in Darora can be defined as tropical (Aw) according Köppen classification (Alvares et al. 2014), with average annual temperature of 27.8 °C, and average annual rainfall of ~1,650 mm, with the driest period concentrated between December and March (± 9% annual precipitation), and the wettest period concentrated between May and August (± 70% annual precipitation) (Barbosa 1997, Barbosa et al. 2007). The study included four habitats occurring in the *lavrado* area, which are used by indigenous people from the Darora community: typical savanna (non-forest formation) and three forest environments (riparian forest, forest island and *buritizal* = *Mauritia* palm formation).

## Sampling methods

### Sampling description

**Figure 1.**
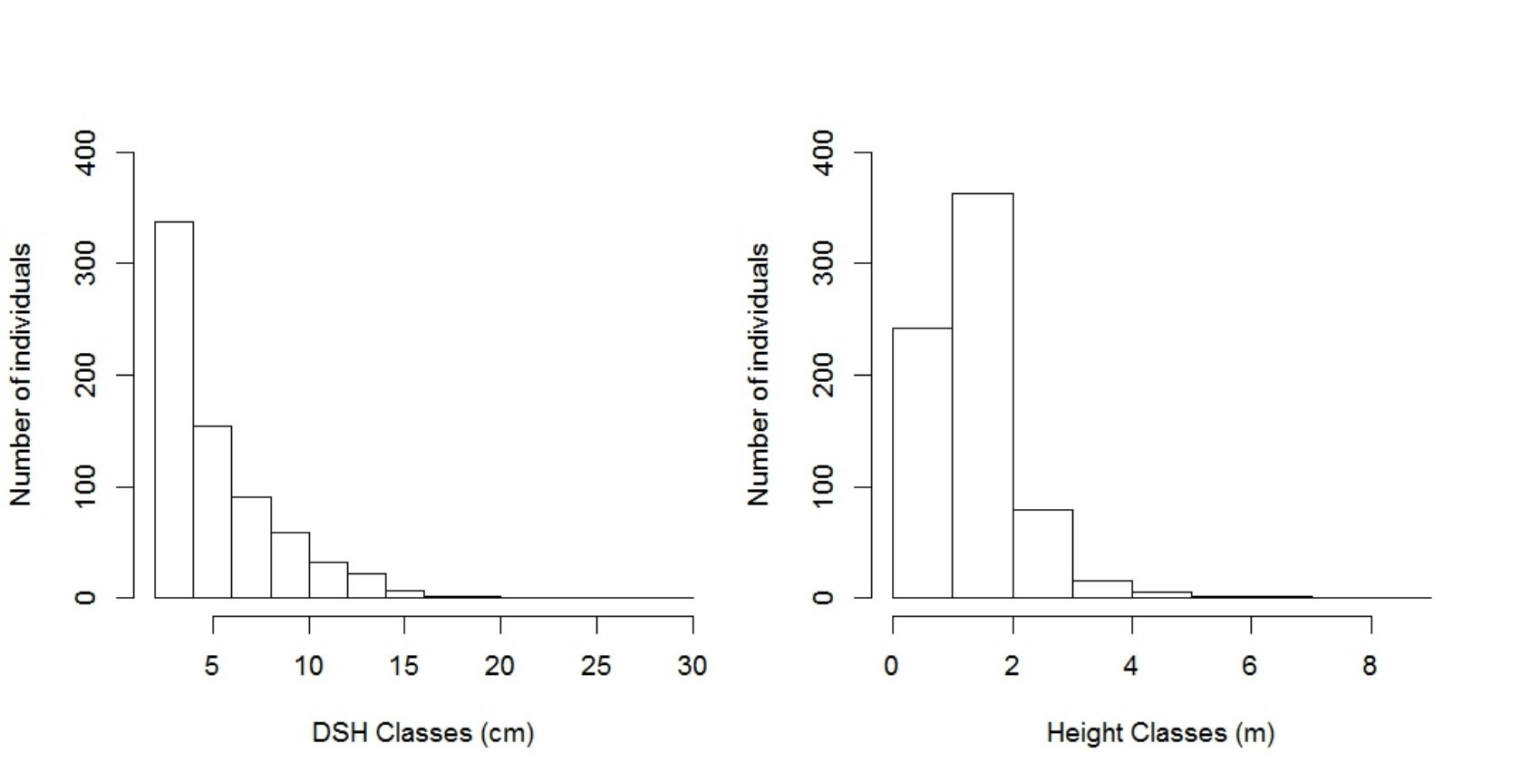
Number of individuals sampled in non-forest habitats in the north of the state of Roraima expressed by diameter classes (DSH ≥ 2 cm); and estimated height of individuals.

Eight plots were installed (each 0.25 ha) at different distances from the Community: four in typical savanna area (non-forest), and another four in several forested habitats (two in riparian vegetation of the Tacutu river, one in a *buritizal* along the Maracajá *igarapé* (stream), and one in an isolated natural forest island) (Fig. 1). Each plot was divided into 10 25m x 10m sub-plots. In the non-forest plots, all trees with diameter greater than or equal to 2 cm, at 2 cm from the soil (DSH _2 cm_ ≥ 2 cm) were measured as suggested by Miranda and Absy (2000) and Barbosa et al. (2005). In the forest plots, all individuals with DBH (diameter at the breast high – 1.3 m) ≥ 10 cm were measured. Additionally, the maximum height of each individual was visually estimated (Suppl. material 1). The Shannon diversity index (H') and Pielou eveness (J') were calculated (Kent and Coker 1995) and the results compared to other studies in Amazonian savanna areas.

Samples of the species were collected, and taxonomic identification was made by expert botanical, parabotanists and local floras (Ribeiro et al. 1999, Melo and Barbosa 2007,Flores and Rodrigues 2010, Wittmann et al. 2010). The nomenclatures were searched in the website of The Plant List (The Plant List 2013). Samples were placed in the herbarium collection of the Universidade Federal de Roraima (UFRR), Boa Vista. Botanical classification followed the APG IV (2016) system. All required federal permissions were obtained (FUNAI: Process 08620.002869 / 2014-15; IPHAN: Process 01450.001678 / 2014-88; CEP-INPA / CONEP: 814370).

## Geographic coverage

### Description

The study area is located in the Darora Community in the São Marcos Indigenous Land, and comprises *ca.* 170 km^2^ (Suppl. material 2). Coordinates: 3°10'42"N and 60°23'34"W.

## Taxonomic coverage

### Description

The study recorded 52 species belonging to 28 botanical families (Table 1). Only 13 species were identified at the genus level. The families with the greatest richness in species (S) were Fabaceae (12 spp.) and Malpighiaceae (5 spp.). Non-forest areas (typical savanna) are the most densely populated by tree individuals (709 stems ha^-1^); however, they presented lower richness (nine species) when compared with typical forest habitats: riparian forest (22 species, 13 families and 202 ind ha^-1^), forest island. (13 species, 10 families and 264 stems ha^-1^) and *buritizal* (19 species, 15 families and 600 stems ha^-1^).

In non - forest habitat, the most abundant species were *Byrsonima crassifolia* (268 stems) and *B. coccolobifolia* (163), while in the forest habitat *Mauritia flexuosa* (27), *Etabalia* sp. (20) and *Curatella americana* (18) were found in greater numbers. *M. flexuosa* dominates *buritizal* areas; however, *C. americana* is a typical species of the non-forest habitat that was densely registered in all the forest environments, especially in the forest island. This is a reflection of the intense extractivism in these environments, enabling several small forest clearings to provide favorable conditions for the recruitment of non-forest species.

*Vertical and horizontal structure*

In non-forest environments, density and basal area were 709 stems ha^-1^ and 2.174 m^2^ ha^-1^, respectively. The diameter was characterized by the predominance of initial classes (DSH _2 cm_ < 5 cm) with a tendency of decrease of individuals in the major classes, in an inverted-J pattern, where the most individuals are distributed in the minor diameter classes while few individuals are found in the major diameter classes (Fig. 1).For vertical structure, most individuals (604) measured up to 2 m in height (Fig. 1), including all individuals of *Byrsonima verbascifolia* and *Palicourea rigida* (both dwarf shrub).

**Figure 2.**
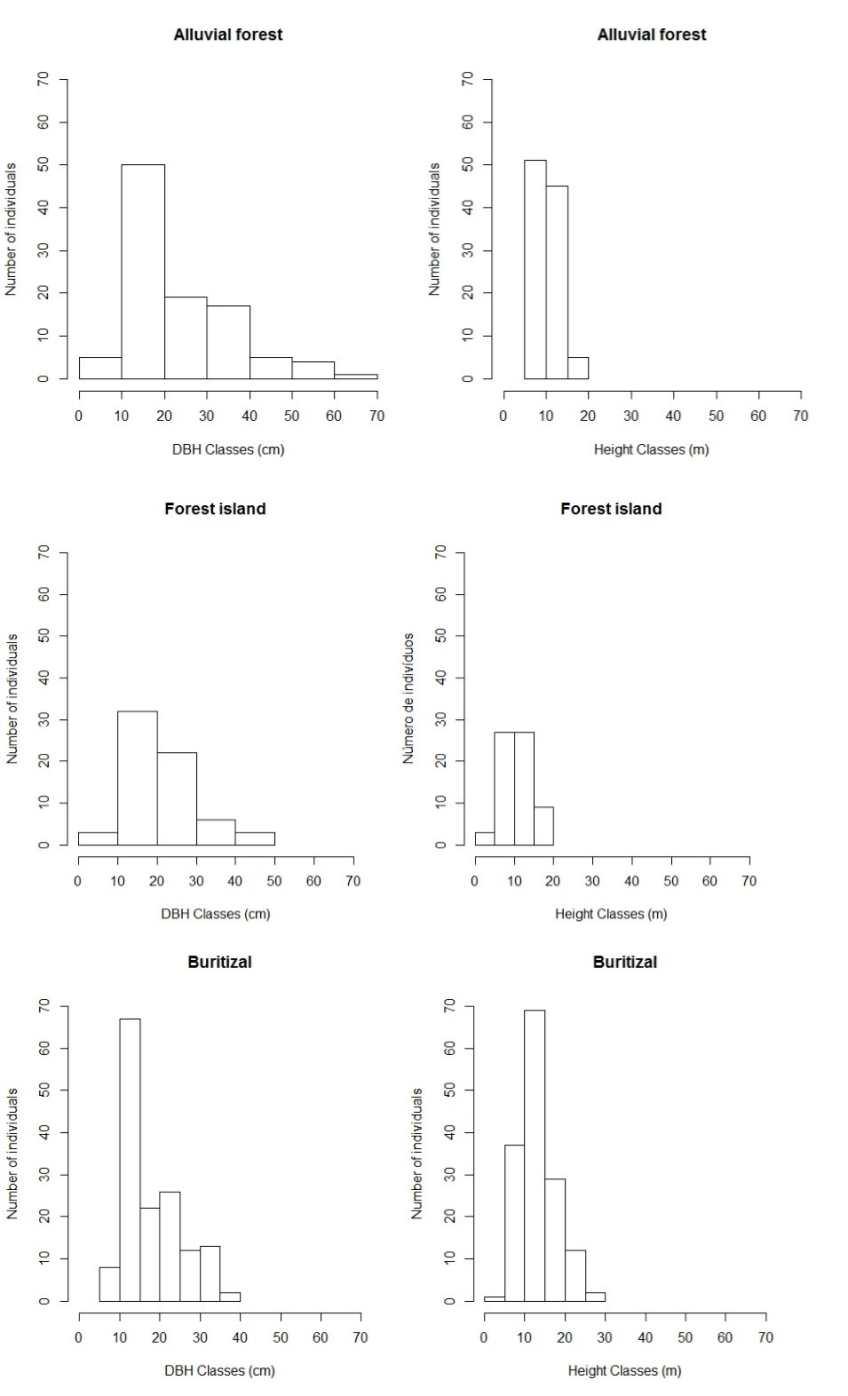
Number of individuals sampled in forest habitats (riparian forest, forest island and *Buritizal*) in the north of the state of Roraima, expressed by diameter classes (DBH ≥ 10cm), and estimated height of individuals.

In forest habitats, the total density was 317 stems ha^-1^, and basal area was 12.41 m^2^ ha^-1^. In *buritizal* habitat, basal area was 4.37 m^2^ ha^-1^, 5.42 m^2^ ha^-1^ in riparian forest and 2.62 m^2^ ha^-1^ in forest island. The distribution of individuals by diameter classes in riparian forest showed that 55 individuals (54%) presented DBH < 20 cm, and 36 (35%) had DBH between 20 and 40 cm, following by a decrease in the major classes, in an inverted-J form (Fig. 2). For vertical structure, the greatest number of individuals (96) occurred between 5 and 15 m in height (Fig. 2). In the forest island, 47 individuals presented DBH between 10 and 30 cm, and 54 individuals (82%) had DBH between 5 and 15 m. In *buritizal* habitat, 97 individuals (64%) presented DBH> 20 cm following a decrease in the major classes. In relation to the vertical structure, 107 individuals (71%) presented height lower than 15 m.

In spite of the differences in the sampling methods and in the criteria for the inclusion of woody individuals, the present results indicated structural and phytosociological similarities with other studies carried out in non-forest (Table 2) and forest (Table 3) formations in savanna areas in the Amazon located in the states of Roraima and Rondônia. The availability of woody resources and the structure of individuals in the sampled areas near the Darora Indigenous Community highlight the need to perform a greater number of floristic inventories in the savanna areas of Roraima. In addition to the impacts represented by the intensive use of woody resources, this large savanna area of northern Amazon has been threatened by the impact of the intense use of natural resources and the rapid expansion of agribusiness and corporate forestry (Aguiar et al. 2014). This threat indicates greater magnitude and a real chance of irreversibility. Therefore, the broad knowledge on plant diversity of the Roraima savanna (*lavrado*) is paramount, and requires a necessary extension of the discussion of public conservation policies for the greatest savanna area of the Amazon biome, as pointed out by Pinto et al. (2014).

## Usage rights

### Use license

Creative Commons Public Domain Waiver (CC-Zero)

### IP rights notes

These data can be freely used, provade resources is cited.

## Data resources

### Data package title

Tree species composition in different habitats of savanna used by indigenous in the Northern Brazilian Amazonia

### Resource link

http://www.gbif.org/dataset/80fdc69b-fb8f-48ef-9066-ade0f60ef5a0

### Alternative identifiers

https://ipt.sibbr.gov.br/sibbr/resource?r=darora_floristic_rr&v=1.12

### Number of data sets

1

### Data set 1.

#### Data set name

Tree species composition in different habitats of savanna used by indigenous in the Northern Brazilian Amazonia

#### Data format

Darwin Core Archive DwC-A

#### Number of columns

25

#### Description

Ocurrences of plants in four habitats in Amazonian Savanna in an indigenous community, State of Roraima. Data set consists of the eml.xml, meta_xml and ocurrence.txt containing the DwC-Attributes.

**Data set 1.** 

Column label	Column description
eventid	A identifier for the record (record code)
institutionCode	Institution that has custody of the object or information about its registration
occurrenceID	A identifier for the occurrence
basisOfRecord	The specific nature of the data record
collectionCode	The name or acronym of the collection or dataset from which the record is derived
catalogNumbe	An identifier (preferably unique) for the record within the dataset or collection
recordedBy	List of names of persons or organizations responsible for the registration of the original occurrence
eventDate	The date or period during which an event occurred
habitat	Description of the habitat in which the event occurred
continent	The Continent of the occurrence
country	The Country of the occurrence
stateProvince	The State or Province of the occurrence
county	The County of the occurrence
locality	The location-specific description
decimalLatitude	The geographical latitude in decimal degrees of the geographical center of a location
decimalLongitude	The geographical longitude in decimal degrees of the geographical center of a location
geodeticDatum	The ellipsoid, geodetic datum, or spatial reference system (SRS) in which the geographical coordinates given in decimalLatitude and decimalLongitude are based
kingdom	Full scientific name of the kingdom in which the taxon is classified
family	Full scientific name of the family in which the taxon is classified
genus	Full scientific name of the genus in which the taxon is classified
specificEpithet	Name of the species epithet of the scientificName
scientificName	The full scientific name. It must be the name of lowest level taxonomic rank that was determined.
identificationQualifier	A brief phrase or standard term ("cf.", "aff.") to express the determiner's doubts about identification.
taxonRemarks	Comments or notes about the taxon or name.
language	Language of the resource.

## Supplementary Material

Supplementary material 1 DBS and Heights of individuals in non-forest (typical savanna) and forest habitats (riparian forest, forest island and *buritizal*) Data type: ocurrences
File: oo_132089.xlsx Rodrigo Leonardo Costa de Oliveira

Supplementary material 2 Ethnomap of Darora Community, Boa Vista, Roraima. Data type: image
Brief description: This ethnomapa was developemented with the participation of the inhabitants of different ages. In legend: Farm area, "roças" (cultivated areas), road, rivers, lakes, residences, frontiers, community center (malocão).
File: oo_157453.jpg Rodrigo Leonardo Costa de Oliveira

## Figures and Tables

**Table 1.** Occurrence of families and species in non-forest (typical savanna) and forest habitats (riparian forest, forest island and *buritizal*). UFRR = number of record in the Herbarium of the Universidade Federal de Roraima.

**Families/Species**	**Typical Savanna**	**Riparian forest**	**Forest island**	***Buritizal***	**UFRR**
ANACARDIACEAE					
*Tapirira guianensis* Aubl.				16	8431
*Spondias mombin* L.		3			-
ANNONACEAE					
*Guatteria* sp.				10	8442
*Xylopia aromatica* (Lam.) Mart.				1	8449
APOCYNACEAE					
*Himatanthus drasticus* (Mart.) Plumel	17		2		8487
*Malouetia* sp.				3	8444
ARECACEAE					
*Mauritia flexuosa* L. f.				27	-
*Euterpe precatoria* Mart.				17	-
BIGNONIACEAE					
*Godmania aesculifolia* (Kunth) Standl.			3		8472
BIXACEAE					
*Bixa arborea* Huber		1			8467
BURSERACEAE					
*Trattinickia rhoifolia* Willd.				7	8440
CARYOCARACEAE					
*Caryocar microcarpum* Ducke				2	8447
CHRYSOBALANACEAE					
*Couepia multiflora* Benth.		1			8450
*Hirtella paniculata* Sw.				2	8435
COMBRETACEAE					
*Buchenavia capitata* (Vahl) Eichler				1	8445
DILLENIACEAE					
*Curatella americana* L.	18	7	18	1	8446
ERYTHROXYLACEAE					
*Erythroxylum suberosum* A. St.-Hil.		2	9		8457
EUPHORBIACEAE					
*Alchornea* sp.				6	8433
FABACEAE					
*Anadenanthera peregrina* (L.) Speg.			4		8475
*Andira* sp.		2			8465
*Bowdichia virgilioide*s Kunth	19		4		8471
*Cassia moschata* Kunth		3			8452
*Cassia* sp.		16			8456
*Copaifera pubiflora* Benth.		2	4		8454
*Cynometra bauhiniifolia* Benth.		8			8460
*Etabalia* sp.		20			8459
*Hydrochorea corymbosa* (Rich.) Barneby & J.W.Grimes		1			8468
*Machaerium aculeatum* Raddi			1		8473
*Ormosia smithii* Rudd.				14	8437
*Swartzia* sp.		3			8478
HUMIRIACEAE					
*Humiria balsamifera* Aubl.				6	8429
LAURACEAE					
*Endlicheria* sp.				14	8432
LECYTHIDACEAE					
*Eschweilera* sp.		3			8463
LOGANIACEAE					
*Antonia ovata* Pohl.	107				8485
MALPIGHIACEAE					
*Bunchosia* sp.		4			8462
*Byrsonima coccolobifolia* Kunth	163				8483
*Byrsonima crassifolia* (L.) Kunth	268		1		8482
*Byrsonima* sp.				3	8443
*Byrsonima verbascifolia* (L.) DC.	19				8480
MALVACEAE					
*Ceiba* sp.		2			-
MORACEAE					
*Sorocea duckei* W.C. Burger		2			8455
MYRISTICACEAE					
*Virola mollissima* (Poepp. ex. A. DC.) Warb.				11	8430
*Virola surinamensis* (Rol. ex Rottb.) Warb.				5	8441
MYRTACEAE					
*Eugenia* sp.		2	6		8464
*Myrcia* sp.		1			8453
PHYLLANTHACEAE					
*Amanoa guianensis* Aubl.				4	8448
PROTEACEAE					
*Roupala montana* Aubl.	81				8484
RUBIACEAE					
*Genipa americana* L.		10	3		8488
*Palicourea rigida* Kunth.	17				8489
SALICACEAE					
*Xylosma benthamii* (Tul.) Triana & Planch.			1		8474
VERBENACEAE					
*Vitex cymosa* Betero ex Spreng.		8			8461
*Vitex schomburgkiana* Schauer			10		8470

**Table 2.** Comparison of richness, diversity and evenness in studies carried out in non-forest habitats occurring in savannas areas of Roraima and state of Rondônia (RO), where: D = diameter used in the research, DBH = diameter at breast height, DSH = diameter at soil height, S = species richness, H' = Shannon diversity index, and J' = Pielou evenness. * Data correspond to a single study that was separately presented here in its different physiognomies to better compare the data.

**Municipality**	**Phytophysiognomy**	**Samplingmethods**	**D**	**S**	**H**'	**J**'	**References**
Boa Vista and Amajari	Savanna tree and Savannah park	Quadrants (80 points) 4 transects	DBH ≥ 5 cm	8	0.8-1.28	0.68-0.80	Sanaiotti 1997
Boa Vista	Savanna grassy-woody and Savanna park	Plots (45 points) 6.75 ha	DSH ≥ 2 cm	71	1.12	0.26	Miranda et al. 2003
Alto Alegre and Boa Vista	Savanna grassy-woody and Savanna park	Plots (3 points) 0.9 ha	DSH ≥ 2 cm	29	0.87	0.26	Barbosa et al. 2005
Vilhena, RO	Cerradão	Plots 1 ha	DBH ≥ 10 cm	60	3.45	0.84	Miranda et al. 2006*
Vilhena, RO	Campo sujo	Plots 1 ha	DBH ≥ 10 cm	26	2.34	0.72	Miranda et al. 2006*
Vilhena, RO	Cerrado *sensu stricto*	Plots 1 ha	DBH ≥ 10 cm	39	2.63	0.72	Miranda et al. 2006*
Vilhena, RO	Cerrado *sensu stricto*	Plots 1 ha	DBH ≥ 10 cm	45	2.9	0.76	Miranda et al. 2006*
Boa Vista	Savanna grassy-woody	Plots (2 points) 1.1 ha	DSH ≥ 6.5 cm	19	0.59-1.2	0.20-0.46	Araújo and Barbosa 2007
Boa Vista	Savanna grassy-woody and Savanna park	Plots (4 points) 1 ha	DSH ≥ 2 cm	9	1.7	0.77	Present study

**Table 3.** Comparison of richness, diversity and evenness in studies carried out in forest habitats occurring in savannas areas of Roraima, where: D = diameter used in the research, DBH = diameter at breast height, DSH = diameter at soil height, S = species richness, H' = Shannon diversity index and J' = Pielou evenness. * Data correspond to a single study that was separately presented here in its different physiognomies to better compare the data.

**Municipality**	**Phytophysiognomy**	**Sampling methods**	**DBH (cm)**	**S**	**H**'	**J**'	**References**
Cantá	Riparian Forest	Plots / 0.4 ha	6	104	6.16	0.92	Sette-Silva 1993*
Cantá	Forest Island	Plots / 0.08 ha	6	47	4.86	0.87	Sette-Silva 1993*
Boa Vista	Riparian Forest	Plots / 0.2 ha	6	59	5.41	0.92	Sette-Silva 1993*
Boa Vista	Forest Island	Plots / 0.28 ha	6	56	4.94	0.85	Sette-Silva 1993*
Cantá	Wooded savanna	Plots 0.35 ha	6	72	4.48	0.73	Sette-Silva 1993*
Cantá	Savanna - SeasonalForest Submontane	Transects (2 points) 3.6 ha	30	61	3.39	0.82	Silva 2003
Mucajaí	Riparian Forest	Plots / 0.4 ha	9.55	33	2.28	0.65	Farias et al. 2012
Boa Vista	Forest Island	Plots (4 points) 0.64 ha	5	52	1.89-3.16	0.67-0.87	Santos et al. 2013
Boa Vista	Forest Island	Plots (12 points) 2.48 ha	10	112	3.86	0.82	Jaramillo 2015
Boa Vista	Riparian Forest	Plots / 0.5 ha	10	22	2.63	0.57	Present study
Boa Vista	Buritizal	Plots / 0.25 ha	10	19	2.59	0.88	Present study
Boa Vista	Forest Island	Plots / 0.25 ha	10	13	2.21	0.86	Present study
