# Supplementary figures and images for: Structure and tree species composition in different habitats of savanna used by indigenous people in the Northern Brazilian Amazon
Source: Biodivers Data J. 2017 Sep 28;(5):e20044. doi: 10.3897/BDJ.5.e20044 (PMC5665001; doi:10.3897/BDJ.5.e20044)

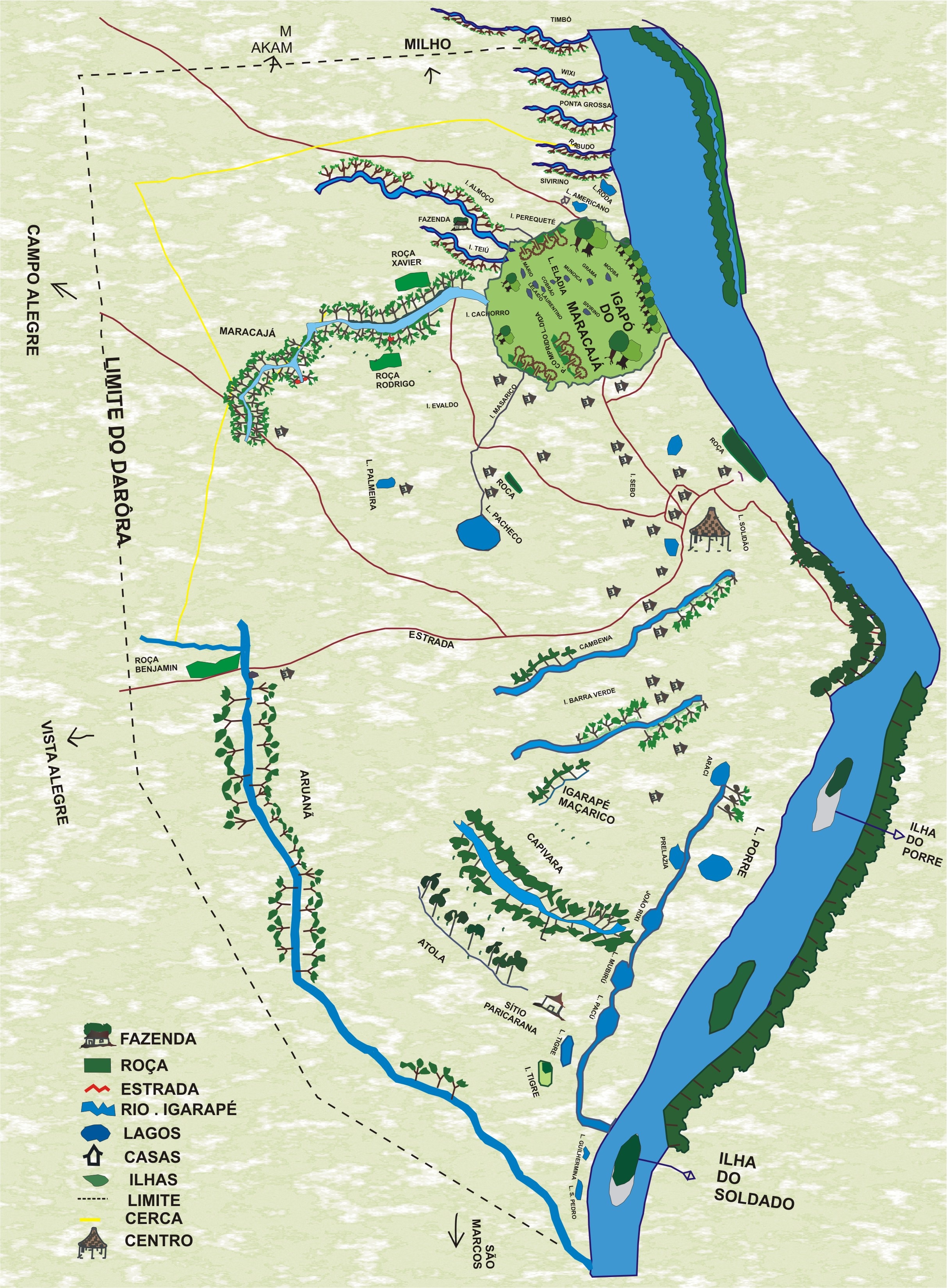

Supplement: Supplementary material 2 — Ethnomap of Darora Community, Boa Vista, Roraima. [file bdj-05-e20044-s002.jpg]
